# Lipopolysaccharide-Preconditioned Periodontal Ligament Stem Cells Induce M1 Polarization of Macrophages through Extracellular Vesicles

**DOI:** 10.3390/ijms19123843

**Published:** 2018-12-03

**Authors:** Hyejong Kang, Myung-Ju Lee, Sang June Park, Myung-Shin Lee

**Affiliations:** 1 Department of Orthodontics, Dankook University Sejong Dental Hospital, Sejong 30107, Korea; hyejongk@dankook.ac.kr; 2 Department of Microbiology and Immunology, Eulji University School of Medicine, Daejeon 34824, Korea; ghostcastle0@gmail.com (M.-J.L.); alclstkdwns@eulji.ac.kr (S.J.P.)

**Keywords:** periodontal ligament stem cell, macrophage, polarization, extracellular vesicles

## Abstract

Periodontitis is a common disease characterized by chronic inflammation and tissue destruction of gums. Human periodontal ligament stem cells (PDLSCs), derived from the periodontium, have stem cell properties similar to those of mesenchymal stem cells. PDLSCs possess not only the potential to differentiate into other tissues, but also immunomodulatory abilities. Macrophages play a critical role in periodontal disease, but little is known regarding the role of PDLSCs in macrophage modulation during inflammation. In this study, we investigated the effect of PDLSCs on the macrophage cell line. While the conditioned media from PDLSCs under normal culture conditions did not affect macrophage polarization, the lipopolysaccharide (LPS)-preconditioned PDLSCs induced significant changes in M1 polarization. Extracellular vesicles (EVs) isolated from the conditioned media of LPS-preconditioned PDLSCs induced strong M1 polarization of macrophages. Additionally, the M1 polarization was abolished by DNase I treatment of EVs. Therefore, the LPS-stimulated PDLSCs induce M1 polarization of macrophages through EVs, suggesting that the EVs from PDLSCs might be a potential therapeutic target for inflammation in the periodontium.

## 1. Introduction

Periodontitis is an inflammatory disease that occurs in response to bacterial infection in the supporting tissues of the teeth. The disease manifests as gingival swelling and bleeding, increased periodontal pocket depth, and alveolar bone loss. Chronic periodontitis is a common disease worldwide, and the prevalence increases with age [1]. The onset of periodontitis is characterized by inflammation of the gingiva in response to a bacterial challenge [2,3]. A previous study indicates that the severity of the disease would have other factors besides the volume of plaque and the bacterial species [4]. The host immune response is a factor in the development of periodontitis, but many aspects of the pathophysiology remain to be elucidated.

Macrophages show plasticity and gain unique phenotype in a different environment. While a pro-inflammatory environment induces an M1 phenotype of macrophage, anti-inflammatory cytokines stimulate an alternative activation of macrophage, termed M2 phenotype [5]. Macrophages play a key role in the inflammatory processes, including periodontitis, as regulators directing inflammation to chronic pathological changes or resolution, with no damage or scar tissue formation by M1 or M2 polarization [6,7,8]. Therefore, the micro-environmental factors that can convert the macrophages into the M1- or M2-type would be important in the development of periodontal disease.

In 2004, periodontal ligament stem cells (PDLSCs) were isolated from the human periodontal ligament [9]. They had the capacity for multipotential differentiation into osteogenic, adipogenic, and chondrogenic lineages in vitro, similar to other human mesenchymal stem cells (MSCs). Since MSCs have been known to be associated with macrophage polarization [10,11], we hypothesized that PDLSCs modulate the polarization of macrophages depending on their microenvironment. During the pathogenesis of chronic periodontitis, the epithelial cells or immune cells respond to bacterial challenge through pattern-recognition receptors (PRRs) and activate the innate immune response [12]. Lipopolysaccharides (LPS) are the primary source of stimulation of PRRs, and they induce the expression of pro-inflammatory cytokines and have received much attention in periodontal disease [8,13,14]. To date, there has been minimal research regarding the role of PDLSCs in the inflammatory microenvironment. In this study, we investigated the role of PDLSCs that were stimulated with or without LPS, in the polarization of macrophages. 

A recent study reported the effect of the conditioned medium from PDLSCs on the periodontal regeneration and mRNA expression of inflammatory cytokines in the murine monocyte/macrophage cell line stimulated with IFN-γ [15]. However, an experimental study on the effect of LPS-preconditioned PDLSCs on macrophages has not, to our knowledge, been reported. Interestingly, we found that the conditioned media from the LPS-preconditioned PDLSCs induced M1 polarization through the extracellular vesicles (EVs), which are released from cells and have been known to be a critical vehicle for intercellular communication [16,17].

## 2. Results

### 2.1. Characterization of Human Periodontal Ligament Stem Cells (PDLSCs)

**Figure 1:**
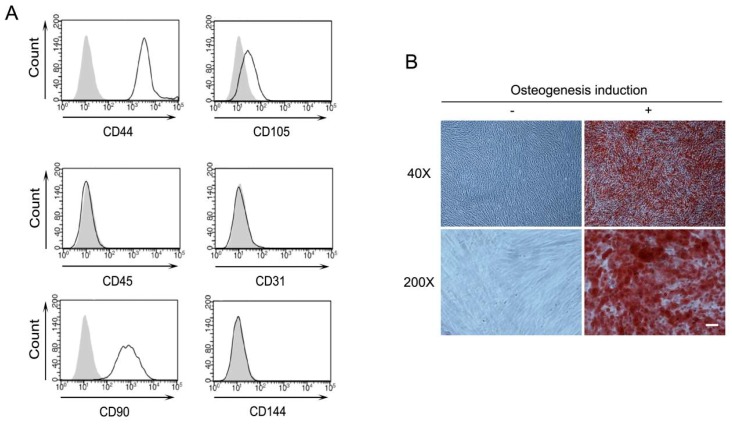
Characterization of periodontal ligament stem cells (PDLSCs). (**A**) Expression of cell surface markers of PDLSCs. Indicated cell surface proteins of PDLSCs were analyzed by flow cytometry. Gray and white areas represent the isotype control and each indicated antibody. (**B**) Osteogenesis of PDLSCs. Mineral deposition after osteogenic differentiation of PDLSCs. Cells were stained with alizarin red S. −: culture media, +: osteogenic differentiation media, magnification, 40× and 200×. Scale bar = 100 μm.

While PDLSCs were certified the surface expression of CD146 and STRO-1 by the supplying company, essential characteristics for mesenchymal stem cells (MSC) were investigated [18]. The immunophenotype of PDLSCs was analyzed by flow cytometry. MSC-associated surface markers, including CD44, CD90, and CD105, were highly expressed on the cell surface of PDLSCs, which is consistent with previous studies in the MSC field [18,19,20,21]. However, the hematopoietic marker (CD45) and endothelial cell markers (CD31 and CD144) were not observed (Figure 1A). In order to assess the differentiation potential, PDLSCs were induced into osteogenic differentiation. After 14–21 days of differentiation, staining of the calcified mineral deposition was observed in the Alizarin Red S staining (Figure 1B).

### 2.2. Effect of the Supernatant from PDLSCs on Macrophage Polarization

**Figure 2:**
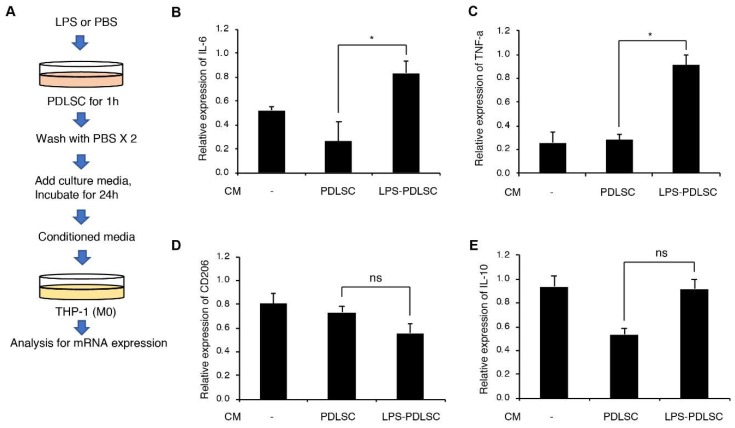
Effect of PDLSCs or LPS-preconditioned PDLSCs on the polarization of the macrophage cell line, THP-1. (**A**) Schematic diagram for the experimental process. M1 or M2 polarization of THP-1 cells by the conditioned media from PDLSCs or Lipopolysaccharide (LPS)-preconditioned PDLSCs. Relative mRNA expression of interleukin (IL)-6 (**B**), Tumor necrosis factor (TNF)-α (**C**), CD206 (**D**), and IL-10 (**E**) were analyzed by RT-qPCR. CM: the conditioned medium from PDLSC and LPS-PDLSC. Data are shown as the mean ± SD, *n* = 3, ns: not significant, * *p* < 0.05.

To investigate if PDLSCs modulate the polarization of macrophages, the supernatant of PDLSCs were added to phorbol 12-myristate 13-acetate (PMA)-differentiated THP-1 cells. Additionally, PDLSCs were pretreated with LPS to evaluate the alteration in the effect of PDLSCs under inflammatory conditions (Figure 2). The conditioned media from PDLSCs did not show any significant effect on the expression of interleukin (IL)-6, tumor necrosis factor (TNF)-α, CD206, and IL-10. These results indicate that PDLSCs under normal culture condition did not modulate the M1 or M2 polarization of macrophages. However, mRNA expression of the M1-associated cytokines, TNF-α and IL-6 in THP-1 cells was significantly increased by the supernatant from LPS-preconditioned PDLSCs. These data suggest that the LPS-preconditioned PDLSCs would affect the M1 polarization of macrophages.

### 2.3. LPS-Preconditioned PDLSCs Accentuate the M1 Polarization of IFN-γ Treated Macrophages

**Figure 3:**
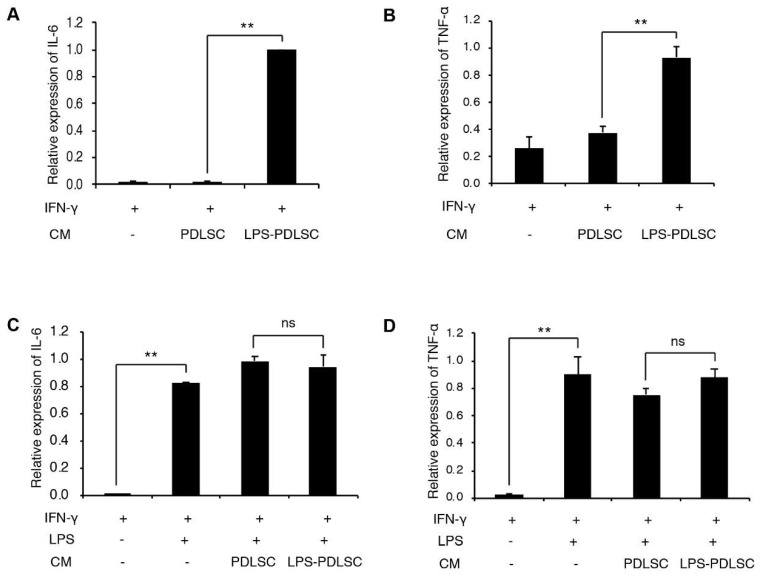
Effect of PDLSCs or LPS-preconditioned PDLSCs on the polarization of THP-1 cells with M1 stimulating agents. The conditioned media from PDLSCs or LPS-preconditioned PDLSCs were added to M0 THP-1 cells with interferon (IFN)-γ (**A**,**B**) or IFN-γ/LPS (**C**,**D**). Relative mRNA expression of IL-6 (**A**,**C**) and TNF-α (**B**,**D**) were analyzed by RT-qPCR. CM: Conditioned medium, IFN-γ: 20 ng/mL of IFN-γ, LPS: 1 μg/mL of LPS, PDLSC and LPS-PDLSC: the conditioned media from PDLSCs and LPS-preconditioned PDLSCs, respectively. Data shown as the mean ± SD, *n* = 3, ns: Not significant, ** *p* < 0.01.

**Figure 4:**
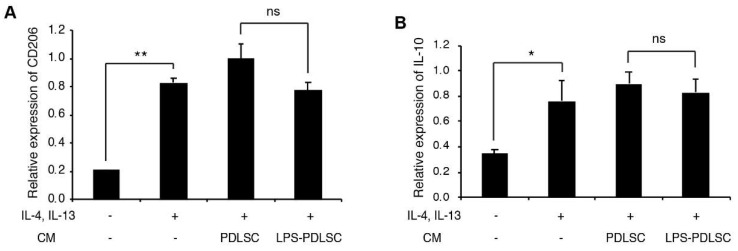
Effect of PDLSCs or LPS-preconditioned PDLSCs on the polarization of THP-1 cells with M2 stimulating agents. The conditioned media from PDLSCs or LPS-preconditioned PDLSCs were added to M0 THP-1 cells with 20 ng/mL of IL-4 and IL-13. Relative mRNA expression of CD206 (**A**) and IL-10 (**B**) were analyzed by RT-qPCR. CM: the conditioned medium from PDLSC and LPS-PDLSC: The conditioned media from LPS-preconditioned PDLSCs. Data shown as the mean ± SD, *n* = 3, ns: Not significant, * *p* < 0.05, ** *p* < 0.01.

To investigate whether the conditioned media from PDLSCs modulate the polarization of macrophages, during M1 or M2 polarization, the conditioned media from PDLSCs were added to the PMA-differentiated THP-1 cells with M1 or M2 stimulating agents (Figure 3 and Figure 4). Interestingly, the conditioned media from LPS-preconditioned PDLSCs showed significantly enhanced M1 polarization effect with IFN-γ, higher than that observed with IFN-γ alone, suggesting that the secreted factors from LPS-preconditioned PDLSCs accentuated the effect on M1 polarization with IFN-γ. LPS-preconditioned PDLSCs did not further enhance M1 polarization on THP-1 cells treated with LPS and IFN-γ simultaneously, which may be because the M1 macrophages had already been fully polarized by the LPS. We also analyzed the effect of the conditioned media from PDLSCs during M2 polarization (Figure 4). mRNA expression of CD206 was slightly decreased by the conditioned medium from LPS-preconditioned PDLSCs, but a significant change in the mRNA expression was not observed for both CD206 and IL-10.

### 2.4. M1 Polarization Was Not Mediated by Free (Soluble) Cytokines from PDLSCs

**Figure 5:**
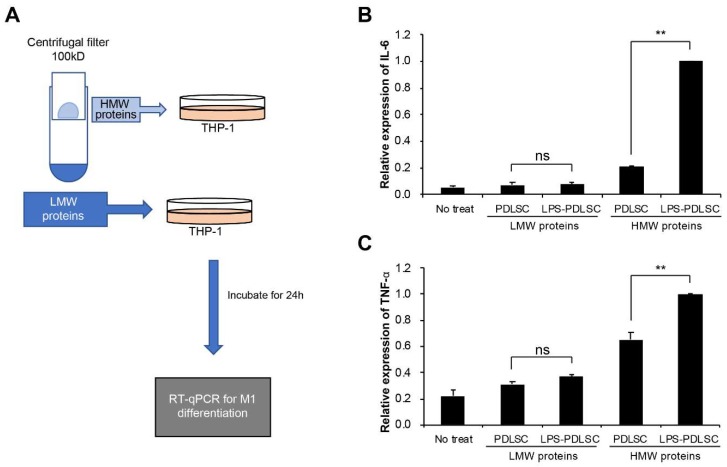
Separation of fractions from the conditioned media of PDLSCs using centrifugal filter device and their effects on the M1 polarization of THP-1 cells. The conditioned media from PDLSCs or LPS-preconditioned PDLSCs were separated using the centrifugal filter device, Amicon ultra-2 100 kDa (Millipore), into high-molecular weight (HMW) proteins and low-molecular weight (LMW) proteins. They were applied to M0 THP-1 cell with IFN-γ and the mRNAs expression for M1 polarization were analyzed. (**A**) Schematic summary of the experimental process. (**B**,**C**) mRNAs expression of THP-1 cells treated with isolated HMW or LMW proteins. Relative mRNA expression of IL-6 (B) and TNF-α were analyzed by RT-qPCR. Data shown as the mean ± SD, *n* = 3, ns: Not significant, ** *p* < 0.01.

To differentiate the effect of small-sized proteins, such as the cytokines, present in the conditioned medium on M1 polarization, the supernatant was separated using a centrifugal filter device with a cutoff at 100 kDa (Figure 5A). Ultrafiltration method have also been used to isolate EVs [22,23,24]. Each separated fraction was added at M0 status of THP-1 cells along with IFN-γ. While no significant difference in M1 polarization was observed between the low molecular weight proteins from the control PDLSCs and LPS-preconditioned PDLSCs, the high molecular proteins containing EVs from the LPS-preconditioned PDLSCs induced a much stronger effect on the M1 polarization of THP-1 cells than the control fraction (Figure 5B,C). These results suggest that EVs from PDLSCs might be a factor for the polarization of macrophage, which supported by recent studies indicating that EVs have a promoting effect for macrophage polarization [25,26]. HMW proteins-treated cells seemed to induce the expression of cytokines compared to non-treated cells, which might be caused by concentrated proteins. Our results demonstrated that the M1 polarization by the supernatant from LPS-preconditioned PDLSCs was not caused by small-sized molecules such as the free (soluble) cytokines.

### 2.5. Isolation of EVs from PDLSCs and Their Characterization

**Figure 6:**
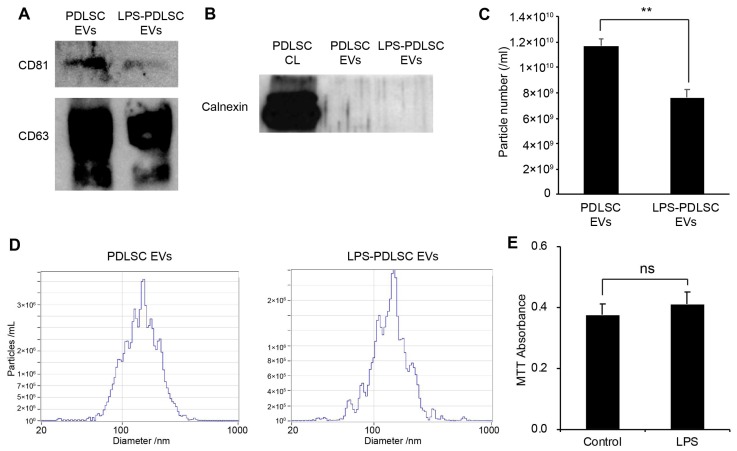
Isolation of EVs from PDLSCs and their characterization. EVs were isolated from PDLSCs by differential centrifugation, as described in the Materials and Methods section. The EVs were analyzed by western blot and nano-particle tracking analyzer, Zetaview. (**A**) Western blot analysis of isolated EVs for the EV markers, CD81, and CD63. (**B**) Western blot analysis of the PDLSC cell lysates and isolated EVs, for the endoplasmic reticulum marker, calnexin. (**C**,**D**). Analysis of particle number and size for each EV sample by Zetaview. (**C**) Particle number of each isolated EV sample. Data shown as the mean ± SD, *n* = 3, ** *p* < 0.01. (**D**) Particle size distribution for each EV sample. (**E**) Cell viability for PDLSCs with or without LPS treatment. Data are shown as the mean ± SD, *n* = 5, ns: Not significant.

EVs were isolated from the same volume of the supernatant of PDLSCs and the LPS-preconditioned PDLSCs by differential centrifugation methods described in the Methods section. While the EV markers, including CD81 and CD63, were detected in EVs from both the control PDLSCs as well as LPS-preconditioned PDLSCs, a lesser amount of the EV markers were observed in the EVs from the LPS-preconditioned PDLSCs, compared to the control PDLSCs (Figure 6A). In order to confirm the contamination of cellular protein in the isolated EVs, the endoplasmic reticulum-specific protein, calnexin, was analyzed in the EVs (Figure 6B). Calnexin was not detected in any of the isolated EVs, suggesting that the EVs were not contaminated with cellular components. The particle number and size of the isolated EVs were analyzed using nano-tracking analyzer, Zetaview (Figure 6C,D). While we used the same volume of EVs from the same conditions, the particle number was significantly decreased in the EVs from LPS-preconditioned PDLSCs compared to those from the control PDLSCs. These results are consistent with the western blot analysis for EV markers. The median particle sizes were 151.3 nm and 146.9 nm for the EVs from control PDLSCs and LPS-preconditioned PDLSCs, respectively (Figure 6D). Our results showed that the concentration of EVs was decreased in PDLSCs by the LPS. To determine whether cell death reduced the EV particles number in the LPS-preconditioned PDLSCs, we analyzed cell viability in the LPS-treated PDLSCs. However, the MTT assay showed similar viability for the control PDLSCs and LPS-treated cells (Figure 6E). 

### 2.6. EVs from LPS-Preconditioned PDLSCs Synergize with the Action of IFN-γ on Macrophages

**Figure 7:**
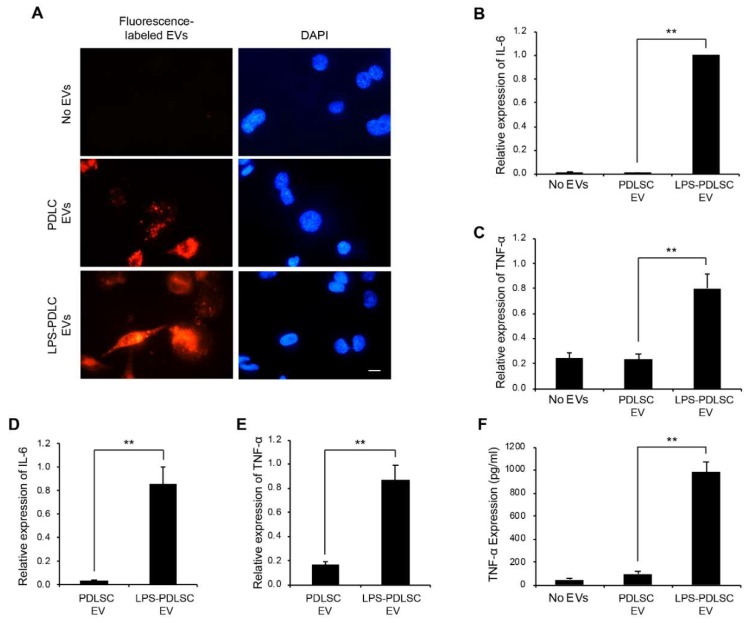
M1 polarization by EVs derived from LPS-preconditioned PDLSCs. (**A**) Entry of EVs into the THP-1 cells. EVs labeled with fluorescence dye were applied to THP-1 cells and their cellular entry was tracked by a fluorescence microscope. PDLSC EVs: EVs from PDLSCs. LPS-PDLSC EVs: EVs from LPS-preconditioned PDLSCs. Scale bar = 10 μm. (**B**,**C**) the M1 polarization of THP-1 cells by EVs from LPS-preconditioned PDLSCs. THP-1 cells were treated with EVs from PDLSCs and LPS-preconditioned PDLSCs, then the mRNA expression of IL-6 (**B**) and TNF-α (**C**) were analyzed by RT-qPCR. (**D**,**E**) M1 polarising effect of EVs from LPS-preconditioned PDLSCs after endotoxin removal. mRNA expression of IL-6 (**D**) and TNF-α (**E**) in THP-1 cells treated with the endotoxin-removed EVs, from PDLSCs and LPS-preconditioned PDLSCs were analyzed by RT-qPCR. (**F**) enzyme-linked immunosorbent assay (ELISA) for TNF-α in the conditioned media from THP-1 cells treated with EVs from PDLSCs or LPS-preconditioned PDLSCs. Data shown as the mean ± SD, *n* = 3, ** *p* < 0.01.

To track the EVs from PDLSCs in the THP-1 cells, the EVs were labeled with a fluorescent dye using ExoGlow-Membrane EV labeling kit (Figure 7A). Both the labeled EVs, from the control PDLSCs and LPS-preconditioned PDLSCs, were detected in the THP-1 cells and we could not find a major difference between them. Since the above result in Figure 3 showed that the conditioned media from LPS-preconditioned PDLSCs strongly enhances the M1 polarization of THP-1 cells, we investigated whether the EVs have a similar effect on macrophages, under the same experimental conditions. After 24 h of EV treatments of THP-1 cells along with IFN-γ, the mRNA expression for IL-6 and TNF-α was analyzed to investigate the M1 polarization of macrophages. The M1 polarization was increased in THP-1 cells treated with EVs from the LPS-preconditioned PDLSCs compared to the control PDLSCs (Figure 7B,C). To exclude the possibility of effects caused by the contaminated LPS, the LPS were removed from the culture supernatant during the EV isolation process, using the endotoxin removal kit. EVs subjected to the endotoxin removal process also showed the M1 polarization effect in THP-1 cells, similar to that of the LPS-preconditioned EVs (Figure 7D,E), indicating that the M1 polarization was not mediated by the residual LPS, but by the EVs. The production of TNF-α was analyzed in EV-treated THP-1 cells by ELISA (Figure 7F). The expression of the TNF-α protein was highly upregulated in the THP-1 cells treated with EVs from the LPS-preconditioned PDLSCs, which is consistent with their mRNA expression.

### 2.7. DNase I Treatment Abolishes M1 Polarization Effect of EVs

**Figure 8:**
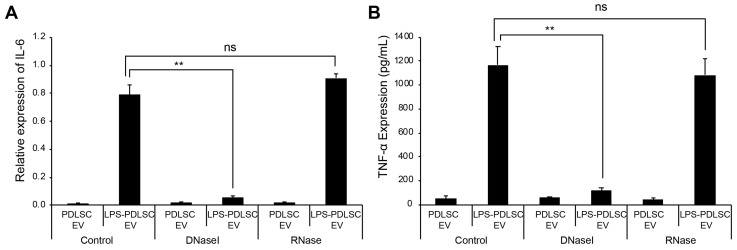
Treatment of EVs with DNase I abolished M1 polarizing effect in THP-1 cells. EVs from PDLSCs or LPS-preconditioned PDLSCs were treated with DNase I or RNase. Each EV sample was applied to THP-1 cells and the M1 polarization was analyzed by RT-qPCR and ELISA. (**A**) mRNA expression of IL-6 in THP-1 cells treated with each indicated EV sample. (**B**) TNF-α expression in the culture supernatant from THP-1 cells treated with each indicated EV sample. Data shown as the mean ± SD, *n* = 3, ns: Not significant, ** *p* < 0.01.

The recent studies showed that the external dsDNA on EVs could be an inducing agent for inflammation [27,28]. To determine whether the DNA or RNA on EVs is associated with the M1 polarization, the isolated EVs were treated with DNase I or RNase, followed by addition to THP-1 cells. Interestingly, only DNase I treatment of EVs significantly abolished the M1 polarization of THP-1 cells, as observed from the mRNA expression data (Figure 8). These results indicate that the external DNA on EVs would be a causative factor for the M1 polarization of macrophages.

## 3. Discussion

Periodontal disease is a common disease afflicting almost half of all Americans over the age of 30 [1,29]. The primary cause of periodontal disease is a plaque with bacterial layers, which causes gum disease and inflammation. The severe periodontal disease leads to unstable teeth or even tooth loss. Previous research suggests that the inflammation associated with the periodontal disease can worsen both diabetes and heart disease condition [30,31]. Various cells are associated with the pathophysiology of the periodontal disease. Among them, the PDLSCs have been highlighted because they are associated with periodontal regeneration and controlling inflammation in the periodontium [32,33]. In this study, we found that the LPS-preconditioned PDLSCs induces the M1 polarization of macrophage through the surface DNA of EVs.

While previous studies showed that the bone marrow or gingiva-derived MSCs induced the polarization of M2 macrophages [11,34,35], there have been few studies on the interrelation between the macrophages and PDLSCs, so far. A previous study showed that the conditioned media from PDLSCs slightly suppressed the mRNA level of TNF-α in murine macrophage RAW 264.7 cells, whereas that of other inflammatory cytokines including IL-6 and IL-1 were not changed [15]. In this study, we isolated the conditioned media from PDLSCs under normal culture or LPS-treated condition and their effect on the polarization of the macrophage THP-1 cells was investigated. Unlike other MSCs, we did not detect M2 polarization of macrophages by the conditioned media from PDLSCs with or without LPS. Intriguingly, the LPS-preconditioned PDLSCs induced M1 polarization of the M0 macrophages and strongly accentuated the M1 activation of macrophages stimulated with IFN-γ. A previous study showed that the LPS-preconditioned human umbilical cord MSCs induce the M2 polarization of macrophages via exosome-shuttled let-7b [36]. Taken together, macrophage polarization might be differently affected depending on the origin of MSCs. Our results demonstrated that EVs play a key role in the M1 polarization of macrophages through their surface DNA, which is consistent with recent studies indicating that inflammatory responses are mediated by secretion of double-strand DNA from EVs [28,37]. To our knowledge, these results are the first demonstration of PDLSCs’ ability to secrete EVs whose surface DNA stimulate an innate immune response.

A recent study indicated that cytokines could be released in EV-encapsulated forms, though they could not be detected by standard cytokine assay [38]. In our experiments, cytokine might be encapsulated in EVs from PDLSCs. However, they do not seem to be a key factor for the M1 polarization of macrophage because DNase I treatment on EVs significantly abolished the polarizing effects.

Generally, inflammation has known to be associated with the innate immune response, against pathogens, through the inflammatory cytokines which have been considered to be primary mediators that control the inflammatory disease. Increasing evidence indicates that EVs play a role as another mediator in cell-to-cell communications via their DNA, RNA, and proteins content [16,17,39]. Since cytosolic DNA works as a strong stimulator of the innate immunity through the cytosolic DNA sensors, including AIM2, cGAS, and TLR3 [27,37], the EV-mediated DNA transport would be an important factor that induces inflammation, independent of the inflammatory cytokines [28,37]. Our results showed that the LPS-preconditioned PDLSCs induced the M1 polarization of macrophages through EVs, suggesting that the EV-bound DNA might be another target to control inflammation in periodontal disease. However, more research is required to elucidate the clinical significance of EVs derived from PDLSCs and the detailed mechanism of M1 polarization by EVs.

In summary, we demonstrated that the EVs from LPS-preconditioned PDLSCs enhance the M1 polarization of the macrophage cell line THP-1. Additionally, we found that the DNA in the outer membrane of EVs would be a critical factor in this response. This study elucidated the relationship between the PDLSCs stimulated with LPS and the polarization of macrophages in vitro, which would provide a new insight into understanding the underlying mechanism of periodontitis.

## 4. Materials and Methods

### 4.1. Cell Culture and Reagents

Periodontal ligament stem cells (PDLSCs) and THP-1 were obtained from Cell Engineering for Origin (Seoul, Korea, http://www.cefobio.com/index.php?hCode=products_02_01_04) and American Type Culture Collection (Manassa, VA, USA), respectively. PDLSC was cultured in α-Minimum Essential Medium (MEM, Gibco, Grand Island, NY, USA) with 10% fetal bovine serum (FBS, Hyclone, Logan, UT, USA) and 1% antibiotics (Lonza, Allendale, NJ, USA). THP-1 cells were cultured in RPMI-1640 medium (Welgene, Seoul, Korea) supplemented with 10% FBS, 0.05 mM of 2-mercaptoethanol, and 1% antibiotics. The cells were maintained in a humidified atmosphere of 5% CO_2_ at 37 °C. LPS from *E. coli* O111:B4 was purchased from Sigma-Aldrich (Gillingham, UK). Recombinant human IL-4 and IFN-γ was obtained from Cusabio technology (Houston, TX, USA). Recombinant human IL-13 was purchased from PEPRO tech (Rocky Hill, NJ, USA). FBS in all experiments was depleted EVs by ultracentrifugation at 100,000 g for 16 h.

### 4.2. Flow Cytometry

PDLSCs were detached by trypsinization. The cells were incubated with fluorescein isothiocyanate (FITC)-labelled antigen-specific primary antibody or FITC-conjugated control IgG (Bethyl Laboratories, Montgomery, TX, USA) for 30 min on ice. FITC-conjugated anti-CD44 antibody (eBioscience, San Diego, CA, USA), FITC-conjugated anti-CD105 antibody (Millipore, Bedford, MA, USA), FITC-conjugated anti-CD90 antibody (eBioscience), FITC conjugated anti-CD45 antibody (eBioscience), FITC-conjugated anti-CD31 antibody (eBioscience) and FITC-conjugated anti-CD144 antibody (eBioscience) were used as primary antibody. After washing, the cells were suspended in 1% FBS/PBS and analyzed using a Guava easyCyte Flow Cytometer and the InCyte 3.1 software (Merck Millipore, Bedford, MA, USA).

### 4.3. Osteogenic Differentiation of PDLSCs

Osteogenic differentiation for PDLSCs was conducted followed by previous studies with some modifications [40,41]. Osteogenic differentiation media (α-MEM containing 10% FBS, 200 mM L-glutamine, 1% penicillin/streptomycin, 10 nM dexamethasone, 50 μg/mL ascorbic acid, 5 mM β-glycerophosphate, and 1.8 mM monopotassium phosphate) was applied to PDLSCs. Differentiation media were changed every 3 days for 14–21 days. Osteogenic differentiation was analyzed by Alizarin red staining.

### 4.4. Quantitative Real-Time Reverse Transcription PCR (RT-qPCR)

Total RNA from cells was isolated by Ribospin II using protocol recommended by the manufacturer (GeneAll, Seoul, Korea). Total RNA was reverse-transcribed to obtain the first-strand cDNA using the PrimeScript 1st stand cDNA synthesis kit (Takara, Otsu, Japan). Real-time PCR was performed using the SYBR^®^ FAST qPCR mix (Takara, Otsu, Japan). The cycling conditions were as follows: 95 °C for 30 s, 40 cycles of 95 °C for 5 s, and 60 °C for 10 s. Specificity of the amplified products was confirmed by analyzing the melting curves. All samples were tested in triplicate using glyceraldehyde-3-phosphate dehydrogenase (GAPDH) as a control. The primers were synthesized by GENOTECH (Daejeon, Korea) and the following primers were used: IL-6s: 5′-GGATTCAATGAGGAGACTT-3′ and IL-6as: 5′-ATCTGTTCTGGAGGTACT-3′ for human IL-6; TNFs: 5′-TATGAGCCCATCTATCTG-3′ and TNFas: 5′-AATGATCCCAAAGTAGAC-3′ for human TNF-α; IL-10s: 5′-TGGAGCAGGTGAAGAATG-3′ and IL-10as: 5′-TCTATGTAGTTGATGAAGATGTC-3′ for human IL-10; CD206s: 5′-AGATGCTGACTGTGTTGT-3′ and CD206 as: 5′-TCGTTGCTGGAGGATTAG-3′ for human CD206; GAPDHs: 5′-GGTATCGTGGAAGGACTC-3′ and GAPDHas: 5′-GTAGAGGCAGGGATGATG-3′ for human GAPDH.

### 4.5. Induction of M1 and M2 Phenotype of THP-1 Cells

Macrophage differentiation and polarization of THP-1 cells were performed as described with modifications [42]. THP-1 cells (2.5 × 10^5^ well) was seeded on 24-well culture plate with 50 ng/mL of phorbol 12-myristate 13-acetate (PMA) and incubate at 37 °C for 48 h. After differentiation into M0, culture media was changed and incubated for 24 h for resting. To induce M1 polarization, LPS (1 μg/mL) and IFN-γ was added to M0 cells and incubate for 24 h. For M2 polarization, IL-4 (20 ng/mL), and IL-13 (20 ng/mL) used and incubated for 66 h.

### 4.6. Isolation of EVs from PDLSCs

**Figure 9:**
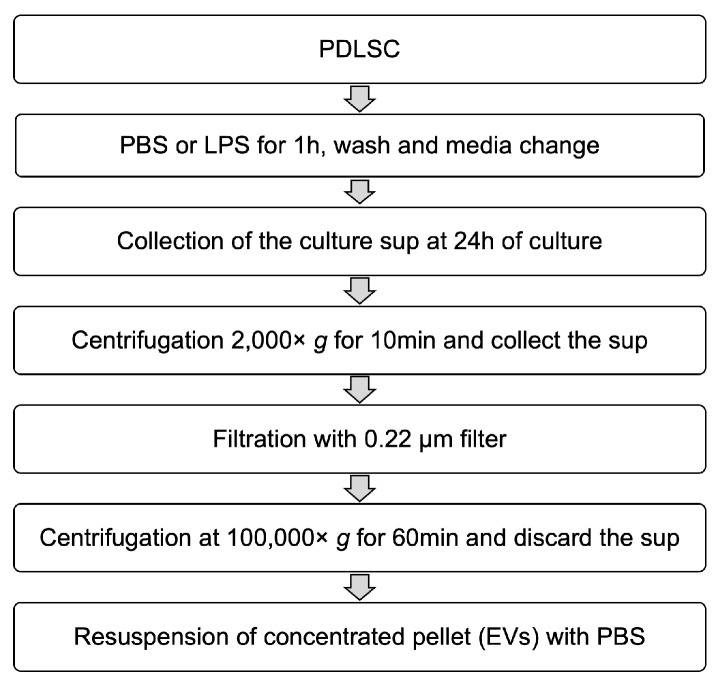
Schematic diagram for the isolation of EVs.

A total of 1 × 10^6^ cells was seeded in 75 cm^2^ cell culture flasks with α-MEM containing 10% FBS and cultured overnight. Phosphate-buffered saline (PBS) or 1 μg/mL of LPS was added to the culture media and the cells were incubated for 1 h. After stimulation, the cells were washed twice with PBS. Then the media was replaced again with fresh culture media. The same volume of each conditioned medium was collected after 24 h of incubation and filtered through a 0.22-μm syringe filter to remove the large extracellular vesicles. Each conditioned medium was applied to THP-1 cells or used for isolation of EVs. To isolate EVs using the centrifugal filter device, Amicon ultra-2 100 kDa (Millipore) was used according to the manufacturer’s instruction. The flowthrough containing low molecular proteins was collected and the high molecular proteins were re-suspended with PBS. For differential centrifugation, the collected media were centrifuged at 2000× *g* for 10 min at 4 °C to remove the cells and debris, followed by filtration through a 0.22-μm filter. The filtered supernatants were transferred to clean ultracentrifuge tube and centrifuged at 100,000× *g* for 60 min at 4 °C and the pellet was re-suspended in cold PBS. The schematic summary is presented in Figure 9.

### 4.7. Nano-Particle Tracking Analysis

The particle size and numbers of nano-particles in the EV preparations were analyzed by the nano-particle tracking analyzer, ZetaView (Particle Metrix GmbH, Meerbusch, Germany). Preparations of EVs were diluted in PBS and passed through 0.22 µm filter before the analysis. The analysis parameters were as follows: Maximum size 200, minimum size 20, brightness 20, sensitivity 75, and temperature 25 °C.

### 4.8. Labeling and Tracking of the Entry of EVs

EVs were stained with ExoGlow-membrane EV labeling kit (System Biosciences, Pala Alto, CA, USA) according to the manufacturer’s instructions. After labeling, the EVs were mixed with 10 ml of PBS and the EVs were isolated again by centrifugation at 100,000× *g* for 1 h. Cells were seeded onto a microscope cover glass. After culturing overnight, the culture media containing the fluorescence-labelled EVs were treated for 3~24 h. The cells were fixed with 4% paraformaldehyde in PBS. After washing, the nuclei were stained using 4,6-diamidino-2-phenylindole and mounted in Vectashield^®^ (Vector Laboratories Inc., Burlingame, CA, USA). Images were observed under the Nikon Eclipse E400 microscope (Nikon Instruments Inc., Melville, NY, USA) using a Nikon Digital site DS-U2 (Nikon Instruments Inc.), and analyzed using NIS element F (version 4.6, Nikon Instruments Inc.).

### 4.9. Remove of Endotoxin from EVs

The isolated EVs from the supernatant of PDLSCs by differential centrifugation was applied to ToxinEraser Endotoxin Removal Kit (GenScript, Piscataway, NJ, USA), as recommended by the manufacturer’s instruction. After the removal process, EVs were centrifuged at 100,000× *g* for 60 min at 4 °C and the pellet was re-suspended in cold PBS.

### 4.10. TNF-α ELISA

Concentrations of TNF-α in the conditioned medium from THP-1 cells were measured by using the human TNF-α ELISA kit (Peprotech, Rocky Hill, TX, USA) according to the manufacturer’s instructions.

### 4.11. Statistical Analyzes

Each experiment was performed at least three times independently, and the representative results are shown. The number of replicates is indicated in each figure legend as “*n*”. Results are shown as the means ± standard deviations. The two-tailed Student’s *t*-test was used to assess the significance of a difference between groups. Statistical significance at *p* values of < 0.05 and < 0.01 is indicated by * and **, respectively.
